# Assessing the links among environmental contaminants, endocrinology, and parasites to understand amphibian declines in montane regions of Costa Rica

**DOI:** 10.1371/journal.pone.0191183

**Published:** 2018-01-11

**Authors:** Christopher J. Leary, Hannah F. Ralicki, David Laurencio, Sarah Crocker-Buta, John H. Malone

**Affiliations:** 1 University of Mississippi, Department of Biology, University, Mississippi, United States of America; 2 University of Connecticut, Department of Ecology & Evolutionary Biology, Storrs, Connecticut, United States of America; 3 Auburn University Museum of Natural History, Department of Biological Sciences, Auburn, Alabama, United States of America; 4 University of Connecticut, Institute of Systems Genomics and Department of Molecular and Cell Biology, Storrs, Connecticut, United States of America; University of South Dakota, UNITED STATES

## Abstract

Amphibians inhabiting montane riparian zones in the Neotropics are particularly vulnerable to decline, but the reasons are poorly understood. Because environmental contaminants, endocrine disruption, and pathogens often figure prominently in amphibian declines it is imperative that we understand how these factors are potentially interrelated to affect montane populations. One possibility is that increased precipitation associated with global warming promotes the deposition of contaminants in montane regions. Increased exposure to contaminants, in turn, potentially elicits chronic elevations in circulating stress hormones that could contribute to montane population declines by compromising resistance to pathogens and/or production of sex steroids regulating reproduction. Here, we test this hypothesis by examining contaminant levels, stress and sex steroid levels, and nematode abundances in male drab treefrogs, *Smilisca sordida*, from lowland and montane populations in Costa Rica. We found no evidence that montane populations were more likely to possess contaminants (i.e., organochlorine, organophosphate and carbamate pesticides or benzidine and chlorophenoxy herbicides) than lowland populations. We also found no evidence of elevational differences in circulating levels of the stress hormone corticosterone, estradiol or progesterone. However, montane populations possessed lower androgen levels, hosted more nematode species, and had higher nematode abundances than lowland populations. Although these results suggested that nematodes contributed to lower androgens in montane populations, we were unable to detect a significant inverse relationship between nematode abundance and androgen level. Our results suggest that montane populations of this species are not at greater risk of exposure to contaminants or chronic stress, but implicate nematodes and compromised sex steroid levels as potential threats to montane populations.

## Introduction

Amphibians are declining on a global scale and at an unprecedented rate [1–7]. An especially puzzling aspect of this phenomenon involves declines of populations in remote, protected areas [1]. For example, amphibians inhabiting relatively undisturbed montane riparian zones in the Neotropics appear to be particularly vulnerable to decline, but why these populations are more severely impacted is unclear [2, 8].

One potential explanation for such “enigmatic” declines [2] integrates global climate change with contaminant exposure, endocrine physiology, and disease [9–21]. For example, the worldwide increase in precipitation, stemming from global warming [22–24], potentially promotes the deposition of environmental contaminants at high elevations via “cold condensation” following volatilization in warm regions [25, 26]. Indeed, alarming levels of contaminants (i.e., organochlorine compounds, organophosphates, carbamates) are increasingly documented in otherwise pristine, high elevation habitats [11, 25, 26]. These effects are of major conservation concern because increased exposure to contaminants can initiate physiological responses that compromise population viability and the health of ecosystems [27–30]. In particular, exposure to contaminants potentially promotes chronic elevations in circulating stress hormone levels (i.e., glucocorticoids)[31–33] that can suppress immune responses and/or sex steroid production [34–37] resulting in increased susceptibility to pathogens and/or reduced reproduction [13, 17, 20, 38–41].

The threat of contaminants to montane amphibian species is well established from work in the Sierra Nevada mountains of the western USA [11, 15, 42–45]. Persistent organic pollutants and “current use pesticides” transported by wind or captured in water evaporating from California’s Central Valley accumulate in clouds, condense at high elevations, and are deposited with precipitation [11, 46–48]. Declining amphibian species inhabiting the Sierra Nevada mountain range possess high concentrations of agricultural pesticides and industrial pollutants originating from upwind sources and surrounding lowland regions [11, 42–44, 49]. Individual contaminant levels often exceed those found in water and soil, indicating that they are accumulating in tissues [50]. In addition to the Sierra Nevada mountain range, the European Alps, the Himalayas, and neotropical montane regions are predicted to be particularly susceptible to contamination by man-made chemicals because of prevailing wind and rainfall patterns and close proximity to dense human populations with high agricultural and industrial activity [51, 52].

Exposure to environmental contaminants can severely perturb the endocrine physiology of wildlife species [28, 31, 53–59] with increasing evidence linking contaminants and endocrine stress to amphibian declines [13, 17]. The vertebrate endocrine stress response involves the production of adrenal glucocorticoids that are regulated by hormones produced by the hypothalamus (corticotrophin releasing hormone, CRH) and anterior pituitary gland (adrenocorticotropic hormone, ACTH). The production of glucococorticoids can be triggered by any environmental perturbation that potentially disrupts homeostasis, including exposure to noxious chemicals [32]. Chronically elevated glucocorticoid levels can repress T and B immune cell proliferation [60] and inhibit the production of sex steroids regulating reproductive behavior [35, 61–63], suggesting that endocrine stress could contribute to amphibian declines by increasing susceptibility to pathogens or decreasing reproduction. Consistent with this hypothesis, exposure to pesticides has been shown to elicit elevations in circulating stress hormones that cause immunosuppression via damage to the thymus gland in African clawed frogs [13, 17] and glucocorticoid administration rapidly inhibits amplexus (mounting) behavior in male newts [64] and vocalization in male frogs [65–66]. Hormonal effects on behavior could also influence the risk of parasitism. For example, testosterone-mediated increases in territorial behavior and reductions in feeding behavior potentially increase ectoparasite loads and decrease endoparasite loads in male mountain spiny lizards, *Sceloporus jarrovi* [67].

The potential links among environmental contaminants, endocrine stress, and immune responses have clear implications for emerging infectious diseases that have been increasingly linked to amphibian declines [68]. While chytridiomycosis, caused by the fungus *Batrachochytrium dendrobatidis* (Bd), has received the most attention [8, 69–73], macroparasites (i.e., nematodes, cestodes and trematodes) can also pose major threats to amphibian populations [19]. For example, the trematode *Ribeoroia ondatrae* causes severe morphological abnormalities that have contributed significantly to amphibian population crashes [72, 74]. Several lines of research link macroparasite infections to environmental contaminants and endocrine disruption in amphibians. For example, exposure to the widely used herbicide atrazine increases trematode infection risks in leopard frogs, *Rana pipiens* [75]. Atrazine has been shown to increase glucocorticoid production in amphibians [17] and glucocorticoid administration can increase susceptibility of amphibians to trematode infections [41], suggesting causal links among contaminants, endocrine stress, and trematode infection risks.

Despite abundant evidence linking declining amphibian populations to contaminants, endocrine disruption, and pathogens, studies that simultaneously assess these factors and the links among them in natural populations are generally lacking, even though integrated approaches may be essential for understanding the complexity of declines [17, 19–21]. Here, we examine environmental contaminants, circulating stress and sex steroid levels, and nematode parasite abundances in high and low elevation populations of the drab treefrog, *Smilisca sordida*, from the Pacific versant of Costa Rica. We focus on a suite of environmental contaminants (i.e., organochlorine, organosphosphate and carbamate pesticides, and benzidine and chlorophenoxy herbicides) that have been implicated in amphibians declines [11, 17]. The goal was to determine whether these variables are interrelated in a way that is indicative of increased threats to montane populations. Specifically, we asked whether high elevation populations were at greater risk of: 1) exposure to environmental contaminants, 2) high circulating stress hormone levels, 3) compromised sex steroid levels, and 4) nematode infections. We found that environmental contaminants were not more prevalent in high elevation populations and that circulating glucocorticoid, estrogen and progesterone levels varied minimally with elevation. However, high elevation populations possessed greater nematode abundances and lower androgen levels that could pose threats to montane populations.

## Materials and methods

### Study species, sites and general procedures

*Smilisca sordida* is a common hylid species found throughout much of Central America and Costa Rica [76]. The species breeds during the dry season in streams [77–79] at elevations ranging from near sea level to 1,525 m [76]. This species is not currently at risk of decline, but its broad geographic and elevational range makes it an ideal subject in which to investigate the relationships among elevation, contaminants, hormones, and pathogens.

**Fig 1:**
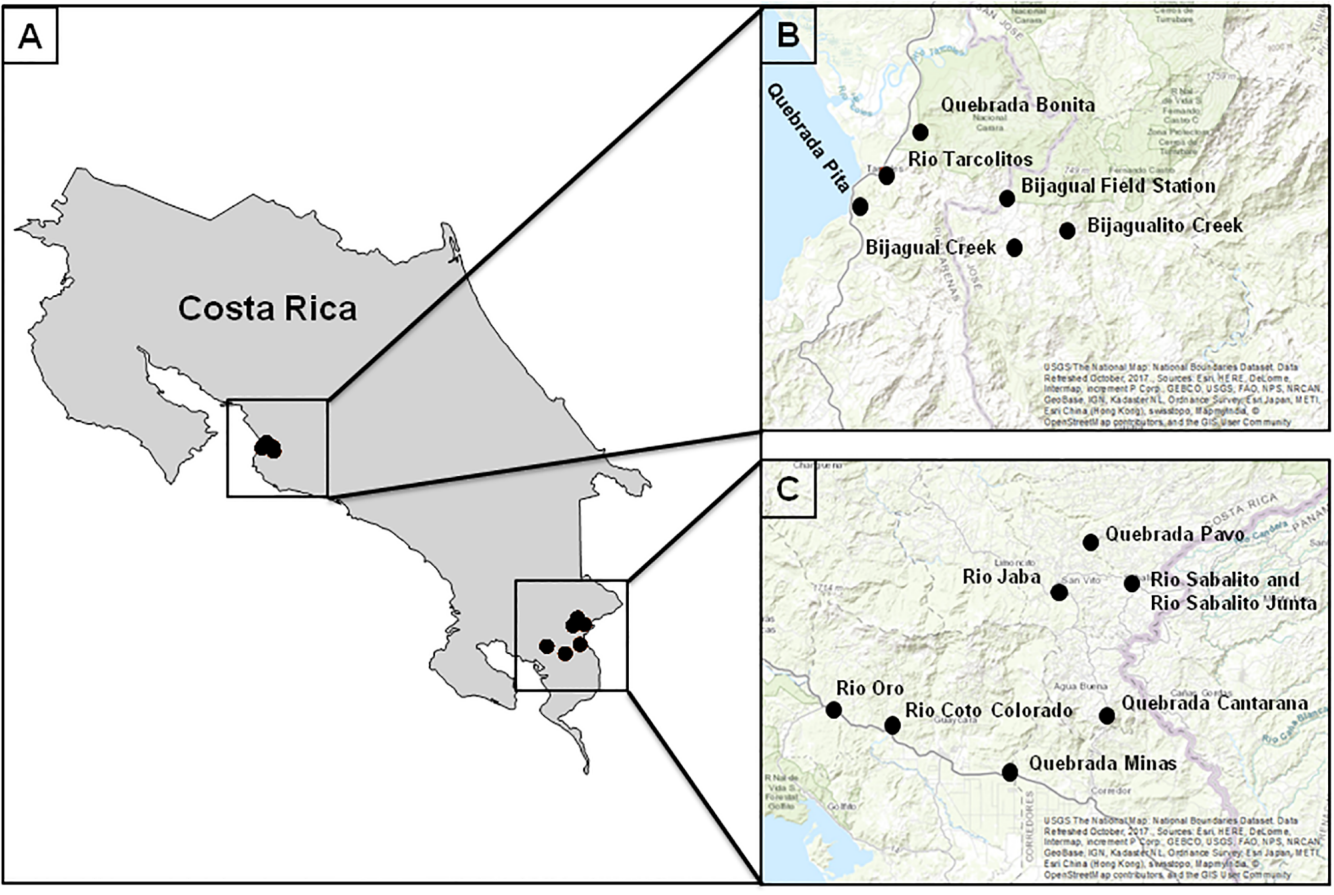
Sampling localities for *S*. *sordida* in Costa Rica. (A) Variation in latitude among sampled populations. (B) Magnified view of northern latitude populations and (C) southern latitude populations. Population elevations and total individuals captured: Bijagual Field Station (449m, n = 10), Bijagual Creek (445m, n = 12), Bijagualito Creek (446m, n = 10), Quebrada Bonita (59m, n = 7), Quebrada Pita (18m, n = 10), Rio Tarcolitos (22m, n = 10), Quebrada Cantarrana (1,053m, n = 7), Rio Jaba (964m, n = 10), Rio Sabalito/Rio Sabalito-Juntas (899m/784m, n = 15), Quebrada Pavo (795m, n = 1), Rio Coto Colorado (76m, n = 16), Quebrada Minas (30m, n = 5), Rio Oro (82m, n = 10).

Populations of *S*. *sordida* were studied in two regions of western Costa Rica ranging from 445–1053 m in elevation for high elevation populations and 18–82 m for low elevation populations (see Fig 1). The northern-most studied region included 6 populations located north of Jaco in and adjacent to the Parque Nacional Carara in Puntarenas and San Jose Provinces consisting of 3 high elevation populations (>445 m) and 3 low elevation populations (<59 m). These populations were characterized by inceptisol soils at low elevations and ultisol/inceptisol soils at high elevations (source: Universidad de Costa Rica, Centro de Investigaciones Agronómicas) and consisted of lowland and premontane tropical moist forests [80]. The southern-most region included 8 populations located north of Golfito in Puntarenas Province consisting of 5 high elevation populations (> 784 m, with only one individual collected from Quebrado Pavo) and 3 low elevation populations (< 82 m)(Fig 1). These populations were characterized by entisol/inceptisol soils at low elevations and andisol soils at high elevations (source: Universidad de Costa Rica, Centro de Investigaciones Agronómicas) and consisted of tropical wet forest and premontane wet forest [80].

Data were collected from male *S*. *sordida* over a period of 8 days during the breeding period from 11–19 March 2016; the narrow time frame of data collection was intended to minimize seasonal variation in circulating hormone levels [81, 82]. Data collection times ranged from 1900 to 0300 h. To minimize the effects that nightly changes in circulating hormone levels [81, 82] could have on comparisons of hormone levels across study sites, nightly sampling periods were randomized across sites (e.g., low elevation sites were not sampled earlier in the evening than high elevations sites or *vice versa*).

Males were found vocalizing at all sites at similar chorus densities. At each site, calling males were captured by hand and rapidly bled via cardiac puncture. Of the 123 males captured, blood was obtained from 119 individuals. Snout-ischial length (SIL) and weight were then measured with a ruler and portable Ohaus digital scale and males were subsequently placed in plastic bags with water and leaves to prevent multiple sampling of the same individuals. Blood samples were immediately transferred to eppendorf tubes and stored on ice until they were centrifuged in less than 8 h after acquisition at 4,000 rpm for 2 min to separate plasma that was immediately frozen.

Of the 123 males captured, 53 (representing all sampled sites) were euthanized within 12 h via rapid decapitation using surgical scissors. The entire liver was then removed and rapidly placed in a liquid nitrogen dewar in labeled cryovials for contaminant analysis. Dissected specimens were then visually inspected for parasites located throughout the abdominal and thoracic cavities and in the gastrointestinal tract. Parasites were removed and placed in ethanol for genetic analysis and identification. Parasites were examined from 52 of the euthanized individuals and adequate blood plasma volume for hormone analysis was acquired from 51 of those individuals. The remaining individuals were released at the point of capture.

Plasma and liver samples were transported back to the United States on ice or in a dry nitrogen dewar. Specimens that were sacrificed for liver samples and contaminant analyses were deposited in the Auburn University, AL, USA Herpetology Collection (Accession numbers: AUM 42955–43008).

### Quantification of contaminants

Contaminant levels in frog liver samples were quantified at the University of Connecticut Center for Environmental Sciences and Engineering. Analysis of tissue contaminants included 31 chemicals commonly used either currently or historically in Costa Rica (i.e., organochlorines and other current use pesticides/herbicides [83]; see S1 Appendix). Each homogenized liver tissue sample (0.2 g) was placed in a micro centrifuge vial, spiked with a surrogate solution and vortex mixed. Acetonitrile (500 uL) was then added and samples were sonicated, vortexed, and centrifuged before being transferred to separate vials. Following extraction, the samples were analyzed with an Agilent (Norwalk, CT, USA) 6890 gas chromatograph equipped with a Restek (Bellefonte, PA, USA) Rxi-5Sil MS column (30m) using splitless injection, coupled to a Waters (Milford, MA, USA) Quattro Micro tandem mass spectrometer (GC/MS/MS). Samples were analyzed for atrazine, 2–4 D, and acetochlor using a Waters ultra-performance liquid chromatograph coupled to a Waters (Milford, MA, USA) Acquity tandem mass spectrometer (UPLC/MS/MS).

All peaks were quantified against an internal standard and extraction efficiency was evaluated using multiple surrogate standards. Standard quality assurance procedures were employed, including analysis of duplicate samples, method blanks, matrix spike duplicates, and laboratory control samples (LCS). Surrogate and LCS percent recovery were all within 85–115%, calibration verification recoveries were all within 90–100%, matrix spike recovery was within 50–130%, and all blanks were below reporting limit. Reporting limits corresponded to the lowest standard on the calibration curve.

### Hormone analysis

Hormone separation and quantification of plasma progesterone (P), dihydrotesterone (DHT), testosterone (T), estradiol (E2), and corticosterone (CORT) concentrations followed the protocol described by [84]. Briefly, plasma samples were incubated overnight with tritiated hormone (PerkinElmer, Inc. Hebron, KY, USA) for determination of recoveries for each sample. Steroids were then extracted from plasma using diethyl ether, dried under nitrogen gas at 40°C, and resuspended in 10% ethyl acetate in iso-octane. Samples were then loaded onto diatomaceous earth columns containing a 3:1 diatomaceous earth:distilled water “glycol trap” and a 1:1 propanediol:ethylene glycol mixture. Mixtures of 2%, 10%, 20%, 40% and 50% ethyl acetate in iso-octane were then used to collect P, DHT, T, E2 and CORT, respectively. Fractions were dried under nitrogen and resuspended in phosphate buffered saline containing 0.3% gelatin for radioimmunoassay. All samples were assayed in duplicate. Antibodies were purchased from the following suppliers: P and T, Fitzgerald Industries International Inc, Acton, MA, USA (#20R-PR053W and #20R-TR018W); E2, ABD Serotec, Raleigh, NC, USA (#7010–2650); CORT, MP Biomedicals, LLC, Solon, OH, USA (#07120016). T antibody was used for both T and DHT assays. All samples were assayed in duplicate and randomized with respect to latitude and elevation across 3 assays. Mean intra-assay coefficients of variation for P, DHT, T, E2 and CORT were 12%, 5.7%, 3.5%, 14% and 12%, respectively, based on 4 standards run with each assay; inter-assay coefficients of variation were 16%, 9%, 6%, 17% and 9%, respectively.

### Parasite identification

Parasites were counted and categorized into four types based on morphology, color, and host tissue location and subsequently preserved in 95% EtOH. We extracted genomic DNA from one parasite type from 2–5 specimens for each of the four designated morphotypes using a DNAeasy kit (QIAGEN, Germany) following manufacturer protocols. To confirm our categorization of unique parasite types and assign genus level identification, we generated an ~800 base pair DNA sequence from the 18S small subunit of rRNA using PCR primers and reaction conditions developed by [85]. The 18S small subunit of rRNA is commonly used as a barcoding gene to aid in the identification of nematode species [86]. Amplification reactions were performed in 10 μl volumes using the following conditions: 2 μl GoTaq 5X Buffer (Promega, Madison, WI, USA); 0.8 μl 1 mM dNTP Mix (Promega, Madison, WI, USA); 0.2 μl 10 μM Nem_18S_F primer (5’-CGCGAATRGCTCATTACAACAGC-3’); 0.2 μl 10 μM Nem_18S_R primer (5’-GGGCGGTATCTGATCGCC-3’); 0.08 μl GoTaq^®^ Polymerase (Promega, Madison, WI, USA); 5.72 μl water; and 1 μl of DNA. Amplification was performed on a BioRad T100 thermocycler (BioRad, Hercules, CA, USA) using the reaction conditions of [85]. Reactions were cleaned using ExoSapIT (Affymetrix, Santa Clara, CA, USA) and clean PCR products were cycle-sequenced using BigDye 3.1 (Life Technologies, Carlsbad, CA, USA) terminator reactions for each sequence direction. Terminator reactions were cleaned with Sephadex size exclusion media (GE Healthcare Life Sciences, Pittsburgh, PA, USA). Clean terminator reactions were sequenced on an ABI 3130 (Life Technologies, Carlsbad, CA, USA) capillary sequencer following standard protocols. Sequence chromatograms were assembled into contigs, edited, and aligned using CLUSTALW in Geneious 8.1.7 (Biomatters Inc., Auckland, New Zealand). Gene alignments were exported as FASTA files for subsequent analyses. Sequences are deposited in Genbank under accession numbers KY856727-KY856739.

We used megaBLAST to compare 18S sequences from collected nematodes to all nucleotide sequences available in Genbank. We used top BLAST hits together with evidence from the literature on nematodes from *Smilisca* [87] to aid in nematode identification. No BLAST hit was a perfect match, most likely because 18S sequence data from nematodes of *Smilisca* were not available in Genbank, and scarce for amphibian hosts in general. Therefore, we regard the genus level identification used throughout the present manuscript as a way to classify the different taxa.

### Body condition

Frogs can lose a significant amount of body mass during the reproductive period [88] and body condition is highly correlated with circulating hormone levels (i.e., CORT) in some anuran species [82, 89]. Hence, body condition estimates were calculated by obtaining the residual values from a linear regression of the cubed root body mass on snout-ischial length (SIL) and dividing those values by the SIL [82]. These values were used to examine the relationships between body condition, hormone levels and nematode abundances.

### Statistical procedures

All variables were first examined for homogeneity of variance and normality. Homogeneity of variance was assessed using Levene’s tests and normality was assessed by determining whether skewness exceeded ± 2 [90]. Total androgen level (DHT + T) and body condition had a skewness within the range of ± 2 which was sufficient to establish normality for univariate analyses [90]. Individual levels of each hormone were non-normally distributed and were logarithmically transformed. A two-way MANOVA was performed to test for latitudinal and elevational differences in circulating hormone levels. The assumption of multivariate normality was tested using a Mardia test [91]. Univariate outliers were examined using boxplots and multivariate outliers were explored using Mahalanobis distances [92]. The assumption of multicollinearity was tested using variance inflation factors (VIF) with a VIF > 10 indicative of robust multicollinearity [93]. Contaminant levels and parasite abundances were non-normally distributed and also contained an excess of zero values. Thus, there was an issue of heteroscedasticity and potential for over-dispersion. Zero inflated models (e.g., negative binomial and Poisson) were inefficient when examining latitudinal and elevational differences in contaminant levels and parasitic abundances across populations. Hence, we coded the presence and absence of contaminant and parasite data with binary values [0 = absent, 1 = present] and ran Fisher’s exact tests. The premise for running Fisher’s exact tests was to correct for low expected values resulting from a bias towards zero. Lastly, we used regression analysis to explore interrelationships among the measured parameters. All analyses were done using SPSS version 24 (IBM software, Chicago, IL USA). To control for multiple comparisons in univariate analyses, a Benjamini–Hochberg correction [94] was implemented using a false discovery rate (FDR) alpha of 0.20.

### Ethics statement

This study was carried out in accordance with the Guidelines for the Euthanasia of Animals by the American Veterinary Medical Association. All procedures were approved by the University of Mississippi Animal Care and Use Committee (protocol #15–015). Scientific collecting permits were granted through El Ministerio de Ambiente, Energia, El Sistema Nacional de Áreas de Conservación, Costa Rica (permit #ACOPAC-INV-008-16).

## Results

### Contaminants

Of the 31 contaminants analyzed (see S1 Appendix), only 9 were detected in the 53 *S*. *sordida* liver samples that were collected. The rank order of the proportion of individuals with detectable levels of contaminants was: aldrin (8 individuals), trans(gamma)-chlordane (6 individuals), dieldrin (3 individuals), 4,4-DDD (3 individuals), trans-nonachlor (2 individuals), cis(alpha)-chlordane (1 individual), cis-nonachlor (1 individual), atrazine (1 individual), acetochlor (1 individual). Of the 26 individuals with detectable levels of contaminants (49% of sampled individuals), only 3 possessed more than one contaminant and none possessed more than two contaminants. Analyses were limited for trans-nonachlor, cis(alpha)-chlordane, cis-nonachlor, atrazine, and acetochlor because they were detected in ≤ 2 individuals and, hence, there were no reliable analyses to test for differences between populations. Results from a Fisher’s exact test indicated that, for all other detected contaminants, there were no differences in the proportions of individuals that tested positive for a particular contaminant in northern versus southern latitudes or high versus low elevation populations (*p* ≥ 0.08; Table 1).

**Table 1:** Results from Fisher’s exact test examining the proportion of *S*. *sordida* possessing contaminants in northern versus southern latitude populations and high versus low elevation populations.

Contaminant	Latitude			Elevation		
	North	South	*p*	High	Low	*p*
	(n = 23)	(n = 30)		(n = 31)	(n = 22)	
*Aldrin*	3	5	1.00	5	3	1.00
*Dieldrin*	0	3	0.25	2	1	1.00
*4*,*DDD*	3	0	0.08	2	1	1.00
*Trans (gamma) chlordane*	2	4	0.69	2	4	0.22

The number of individuals sampled is provided at the top of each column and the number of frogs possessing the specified contaminant is provided in each cell. Analysis was based on a total of 53 frogs.

### Hormone levels

The time of night that blood samples were collected did not differ for northern and southern latitude populations (t- test, *p* = 0.77, n = 119) or low and high elevation populations (t- test, *p* = 0.73, n = 119) and there was no evidence of a negative relationship between any of the measured steroid hormone levels and the duration of time that blood samples were stored on ice before they were centrifuged to separate plasma (*p* ≥ 0.23, n = 119), indicating that steroids did not degrade over this period of time. Blood acquisition time following capture by hand ranged from 60 to 430 s (*mean* = 190 s, *SD* = 73) and was unrelated to circulating progesterone (*F*_1,117_ = 0.65, *p* = 0.42), estradiol (*F*_1,117_ = 1.58, *p* = 0.21), total androgens (*F*_1,117_ = 3.28, *p* = 0.07), or corticosterone level (*F*_1,117_ = 0.028, *p* = 0.89). Blood acquisition time did not differ for northern and southern latitude sites (F_1,117_ = 0.114, *p* = 0.74) or high and low elevation sites (F_1,117_ = 0.004, *p* = 0.95). Hence, all samples were used in data analysis.

Variation in the five measured hormones from each of the northern and southern latitude sites and across elevations is provided in S1 Fig. Kendall’s tau-b correlation analysis revealed strong correlations among unadjusted levels of the measured hormones (see S1 Table). Hence, a two-way MANOVA was used to test for latitudinal and elevational differences in circulating hormone levels. Prior to analysis, all hormonal variables were logarithmically transformed to ensure normality. Despite strong correlations between circulating hormones levels, multicollinearity was not an issue (VIF < 3 for all comparisons). There was an overall main effect of latitude (Wilks’ Lambda: *F*_5, 111_ = 11.69, *p* < 0.0001) and elevation on log-transformed hormone levels (Wilks’ Lambda: *F*_5, 111_ = 3.40, *p* = 0.007) and a significant interaction between latitude and elevation on log-transformed hormone levels (Wilks’ Lambda: *F*_5,111_ = 5.43, *p* < 0.0001).

**Fig 2:**
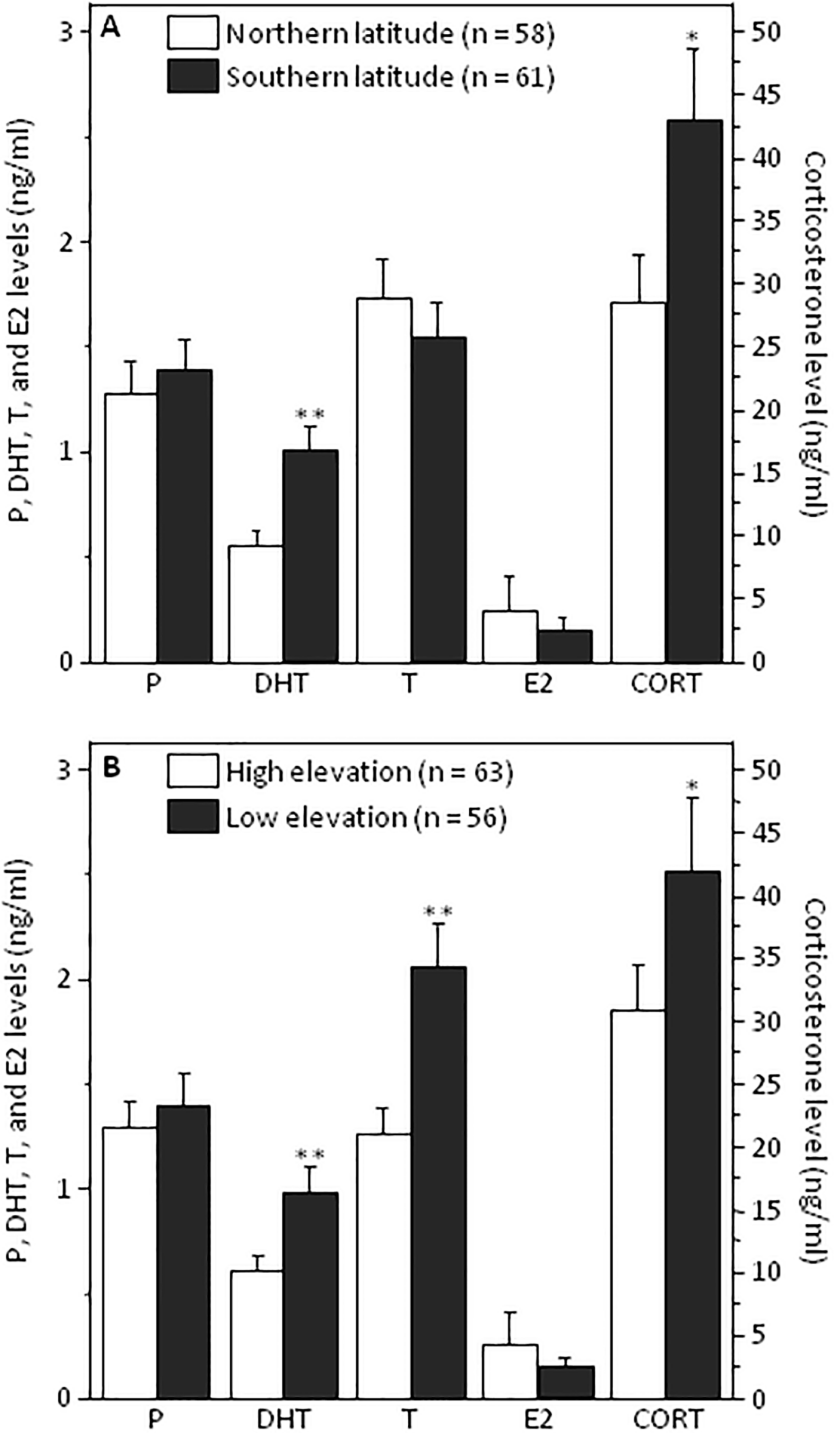
Circulating hormone levels in *S*. *sordida*. Levels of progesterone (P), dihydrotestosterone (DHT), testosterone (T), estradiol (E2) and corticosterone (CORT) in (A) northern and southern latitude populations and (B) high and low elevations. Two asterisks indicate significance with a Benjamini and Hochberg correction and single asterisk indicates significance only when the correction was not applied. Bars represent ±1SE.

To further examine how levels of the five measured hormones varied with latitude and elevation, we used univariate tests with and without Benjamini-Hochberg correction on log- transformed and untransformed data. There was no main effect of latitude on log-transformed levels of P (*F*_1,115_ = 0.56, *p* = 0.45), T (*F*_1,115_ = 0.93, *p* = 0.37), or E2 (*F*_1,115_ = 0.03, *p* = 0.87) (Table 2). However, there was a significant main effect of latitude on DHT (*F*_1, 115_ = 16.80, *p* < 0.0001). There was only a significant main effect of latitude on CORT when a Benjamini-Hochberg correction was not applied (*F*_1,115_ = 4.40, *p* = 0.03). Thus, southern populations had higher circulating levels of DHT and possibly CORT (Fig 2A). Overall results were the same for transformed and untransformed data (Table 2) and thus untransformed data are presented in Fig 2A.

**Table 2:** Two-way MANOVA results comparing hormone levels by latitude and elevation in male *S*. *sordida*.

Hormone	*p*(latitude)	*p*(elevation)	*p*(elevation x latitude)
*Progesterone*	0.45	0.58	0.18)
	(0.43)	(0.62)	(0.07)
*Dihydrotestosterone*	**<0.0001**	**0.006**	**0.006**
	**(<0.0001)**	**(0.004)**	**(0.005)**
*Testosterone*	0.37	**<0.0001**	0.52
	(0.43)	**(0.001)**	(0.34)
*Estradiol*	0.87	0.74	0.95
	(0.63)	(0.50)	(0.54)
*Corticosterone*	0.03	0.03	0.32
	(0.03)	(0.09)	(0.19)

Results using log-transformed data are provided in top row for each hormone and results using untransformed data are provided in second row in parentheses. Bold indicates significance under Benjamini and Hochberg correction (FDR alpha = 0.20). Sample sizes: northern latitude n = 58, southern latitude n = 61, low elevation n = 63, high elevation n = 56.

There was also no main effect of elevation on log-transformed levels of P (*F*_1, 115_ = 0.29, *p* = 0.58) or E2 (*F*_1,115_ = 0.11, *p* = 0.74)(Table 2). However, there was a significant main effect of elevation on DHT (*F*_1,115_ = 7.93, *p* = 0.006) and T (*F*_1,115_ = 14.53, *p* < 0.0001)(Table 2). There was only a main effect of elevation on CORT level when a Benjamini-Hochberg correction was not applied (*F*_1,115_ = 4.93, *p* = 0.03)(Table 2). Results were highly similar for transformed and untransformed hormone data (Table 2). In summary, low elevation populations had higher circulating levels of DHT, T, and possibly CORT than high elevation populations (see Fig 2B for untransformed data). Lastly, the overall interaction effect detected from MANOVA (described above) was attributable to a significant interaction between elevation and latitude on DHT (*F*_1, 115_ = 7.69, *p* = 0.006; Table 2); southern lowland populations had the highest levels of DHT (see S1 Fig).

**Fig 3:**
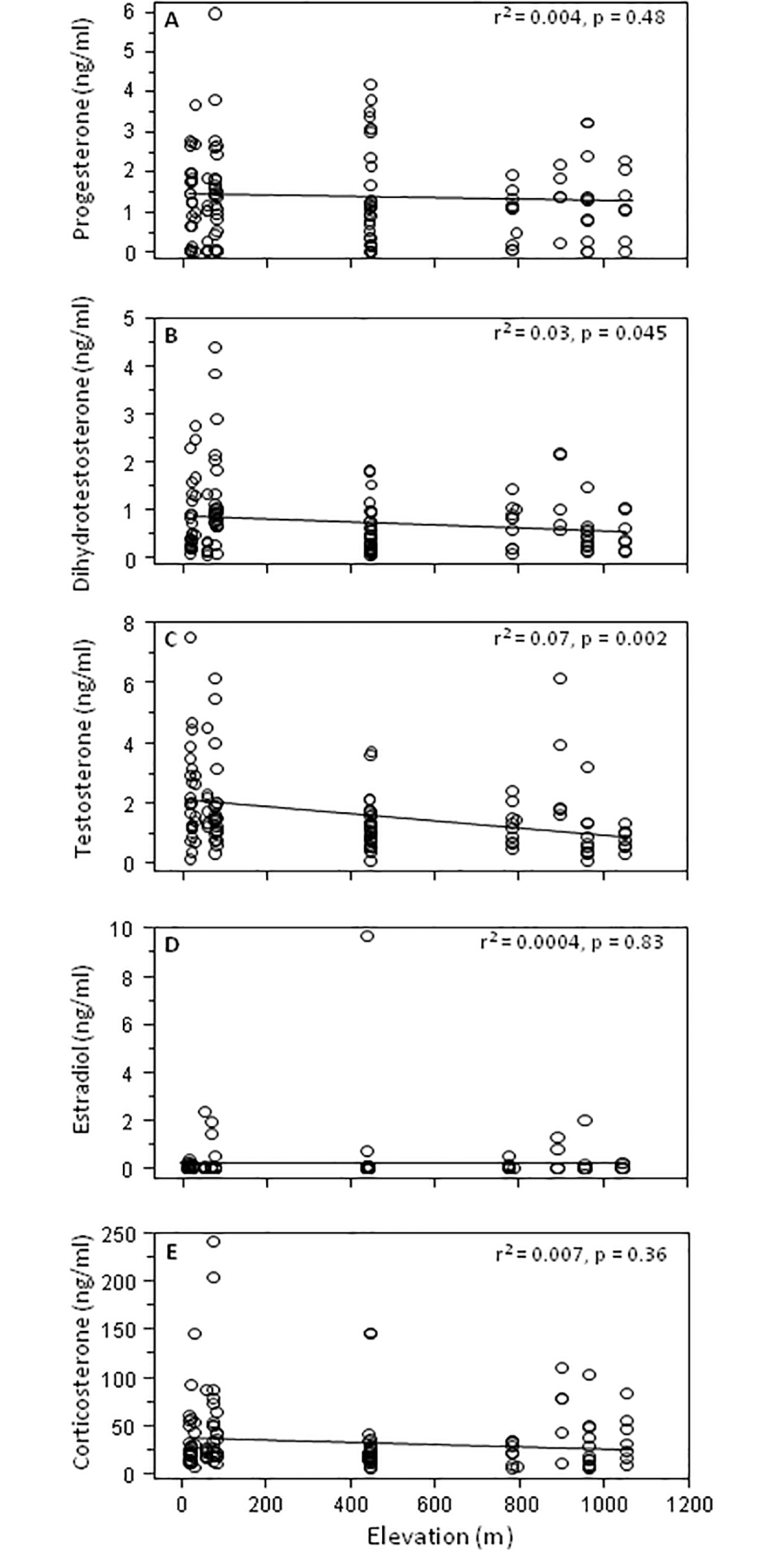
Relationship between log-transformed hormone levels and elevation in *S*. *sordida*. (A) progesterone, (B) dihydrotestosterone, (C) testosterone, (D) estradiol and (E) corticosterone (n = 119).

To further explore the relationships between hormone levels and elevation, we used linear regression on log-transformed and untransformed data which yielded the same overall results and thus, only results for untransformed data are presented. Elevation was not significantly related to circulating levels of P (*r*^*2*^_1,117_ = 0.004, *p* = 0.48), E2 (*r*^*2*^_1,117_ = 0.004, *p* = 0.83), or CORT (*r*^*2*^_1,117_ = 0.007, *p* = 0.36)(Fig 3). However, elevation was negatively correlated with T level whether a Benjamini-Hochberg correction was applied or not (*r*^*2*^_1,117_ = 0.07, *p* = 0.002). Elevation was only negatively correlated with DHT level when a Benjamini-Hochberg correction was not applied (*r*^*2*^_1,117_ = 0.03, *p* = 0.045; Fig 3).

### Parasites

The five top BLAST hits for each parasite sequence are provided in S1 Table. Four nematode genera were identified: 1) *Setaria* sp. (Filarioidea, Setariidae), 2) *Cosmocercoides* sp. (Cosmocercoidea, Cosmocercidae), 3) *Oswaldocruzia* sp. (Trichostrongyloidea, Molineidae), and 4) *Rhabdias* sp. (Rhabditoidea, Rhabdiasidae). Genetic analysis revealed distinct differences in each of the 4 assigned morphotypes but little evidence of sequence variation within each morphotype, indicating that our categorization of nematodes accurately reflected actual nematode diversity and assignment of taxa.

Nematodes were found in 34 of the 52 male *S*. *sordida* (65%) that were examined; males were infected with an average of 7.2 ± 8.46 nematodes (analysis includes uninfected frogs). *Setaria* sp. were the most abundant nematode with an average of 3.69 ± 6.05 individuals found in the body cavity of *S*. *sordida*, followed by *Cosmocercoides* sp. with 2.12 ± 3.82 individuals in the lower intestine, *Rhabdias* sp. with 0.77 ± 3.20 individuals in the lungs, and *Oswaldocruzia* sp. with 0.63 ± 2.0 individuals in the upper intestine. Results from a Fisher’s exact test indicated that there were no differences in the proportion of individual frogs possessing *Setaria* sp., *Cosmocercoides* sp., or *Oswaldocruzia* sp. in northern versus southern latitude populations (*p* ≥ 0.09; Table 3); however, there was a significantly higher proportion of frogs possessing *Rhabdias* sp. in southern latitude populations (*p* = 0.003; Table 3). The proportion of frogs possessing *Setaria* sp., *Cosmocercoides* sp., and *Rhabdias* sp. was higher in high elevation populations (*p* ≤ 0.01); there was no difference in the proportion of frogs with *Oswaldocruzia* sp. in low and high elevation populations (*p* = 0.07; Table 3). *Oswaldocruzia* sp. was not found in any of the frogs sampled at low elevations and *Rhabdias* sp. was not found in any of the northern latitude or low elevation populations (Table 3).

**Table 3:** Results from Fisher’s exact test examining the proportion of *S*. *sordida* possessing *Setaria* sp., *Cosmocercoides* sp., *Oswaldocruzia* sp., and *Rhabdias sp*. in northern versus southern latitudes and high versus low elevation populations.

Parasite	Latitude			Elevation		
	North	South	*p*	High	Low	*p*
	(n = 23)	(n = 29)		(n = 31)	(n = 21)	
*Setaria*	9	18	0.16	22	5	**0.002**
*Cosmocercoides*	6	15	0.09	17	4	**0.01**
*Oswaldocruzia*	2	4	0.68	6	0	0.07
*Rhabdias*	0	9	**0.003**	9	0	**0.007**

The number of individuals sampled is provided at the top of each column and the number of frogs possessing the parasite is provided in each cell. The analysis was based on a total of 52 frogs. All values remained significant after application of Benjamini and Hochberg (FDR alpha = 0.20).

### Body condition

Body condition estimates were normally distributed and were not transformed for analysis. Two-way ANOVA indicated that there was a significant interaction between the effect of latitude and elevation on body condition (*F*_1,119_ = 6.62, *p* = 0.01); males from the northern latitude populations were in poorer body condition (*F*_1, 119_ = 7.15, *p* = 0.009) and low elevation populations were in poorer condition (*F*_1,119_ = 4.65, *p* = 0.03). Importantly, the difference in body condition for low and high elevation populations equated to less than 1 g, suggesting that the greater body condition indices for high elevation populations was attributable to the weight associated with greater nematode abundances. Consistent with this hypothesis, regression analysis indicated that individual total nematode abundance was significantly and positively correlated with body condition estimates (*r*^*2*^_1, 50_ = 0.12, *p* = 0.01).

Because of the potential effect of nematode abundances on body condition estimates, we used individual nematode abundances as a covariate in linear regression to examine the relationship between body condition and circulating hormone levels. Nematode abundance was not a significant factor in regression analyses for any of the 5 measured hormones whether the data was log-transformed or not (*p* ≥ 0.08). Furthermore, body condition was not an accurate predictor of transformed or untransformed hormone levels (*p* ≥ 0.13).

### Interrelationships among parameters

We first performed simple linear regression analysis to examine the relationships among the measured parameters, focusing on variables that differed between high and low elevation populations (total androgen level and total nematode abundance, neither of which were transformed because they were normally distributed). Total and individual nematode abundances were tested for heteroscedasticity using the Breusch-Pagan test [95] and for over-dispersion using Poisson regression. If heteroscedasticity and/or over-dispersion were present, a zero-inflated negative binomial regression was implemented and compared to a standard negative binomial model using the Vuong test [96]. In all cases, the Vuong test found the standard negative binomial model to be a significant improvement over the zero-inflated model and thus we report the results from the standard model.

**Fig 4:**
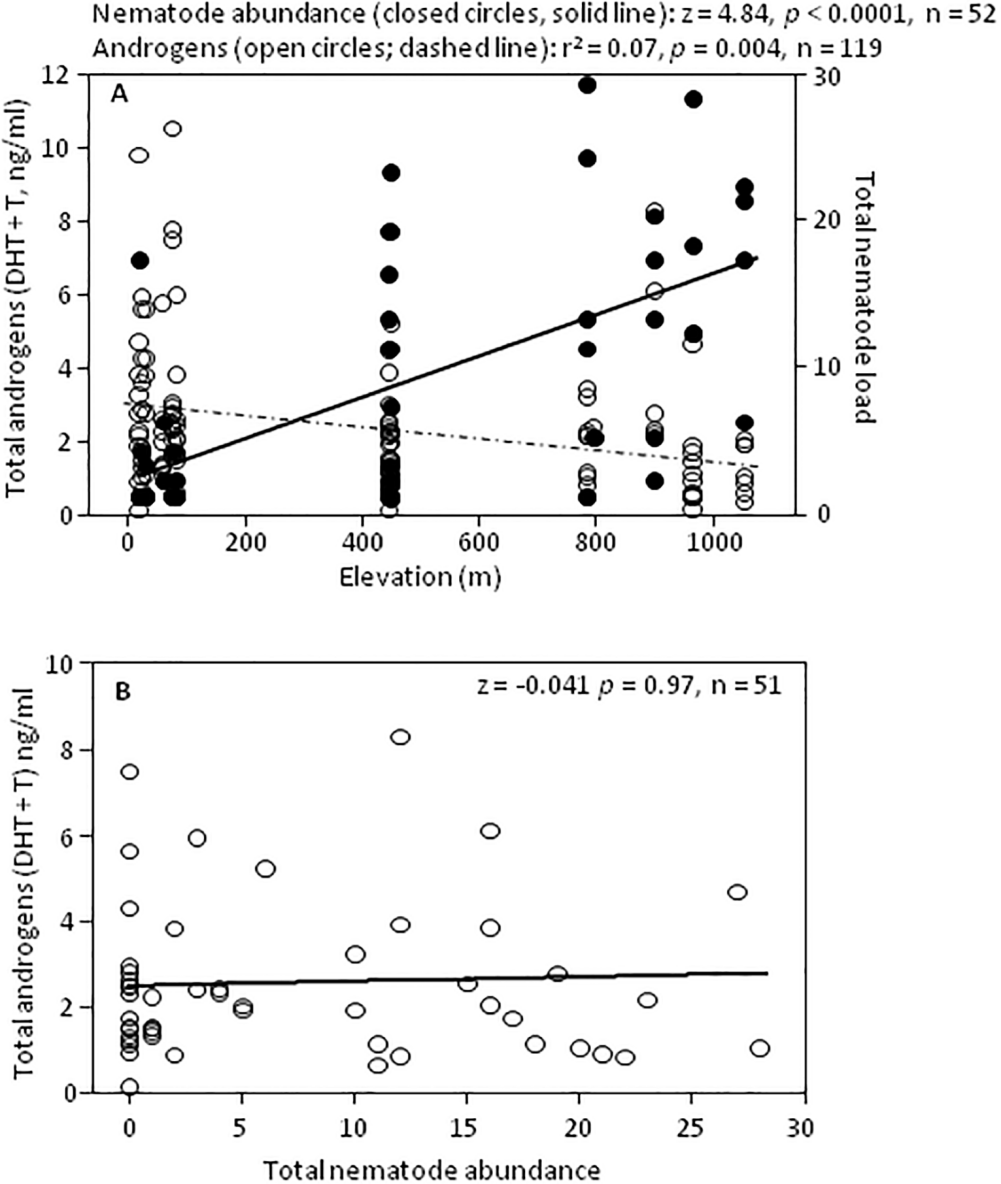
Androgens, elevation and nematode abundance in *S*. *sordida*. Relationships between (A) elevation and total androgen level, and elevation and total nematode abundance, and (B) total nematode abundance and total androgen level.

Elevation was negatively related to total androgen (DHT + T) level (*r*^*2*^_1,117_ = 0.07, *p* = 0.004; Fig 4A) and positively related to total nematode abundance (z = 4.89, *p* < 0.0001; Fig 4A). Thus, there was a significant interaction between total androgens and elevation on total nematode abundance (z = 2.90, *p* = 0.004); high elevation populations had lower androgen levels and higher nematode abundances (Fig 4A) suggesting that high parasite abundances in high elevation populations negatively affected androgen levels. However, total androgen level was not inversely related to total nematode abundance (z = -0.041 *p* = 0.97; Fig 4B). There was also no evidence that infected and uninfected males in each high elevation population differed in total androgen level (unpaired t-tests, *p* ≥ 0.12). Results were similar when DHT and T levels were analyzed separately (data not shown).

Next, we performed regression analyses to examine the relationships among elevation, total androgen level (untransformed), and nematode species abundances. The abundance of nematodes was not significantly related to total androgen level for *Setaria sp*. (z = -0.45, *p* = 0.65), *Cosmocercoides sp*. (z = 1.17, *p* = 0.26), *Oswaldocruzia sp*. (z = 0.06, *p* = 0.94) or *Rhabdias sp*. (z = -0.17, *p* = 0.86).

While circulating levels of CORT were not significantly predicted by elevation (Fig 3E), there was some, albeit weak, evidence that CORT differed between high and low elevation populations (Fig 2B). Hence, we also examined the relationship between CORT level and nematode abundance. Regression analysis indicated that total nematode abundance did not significantly predict log-transformed CORT levels (z = 0.35, *p* = 0.73) or untransformed CORT values (z = 0.42, *p* = 0.67).

## Discussion

The interrelationships among environmental contaminants, pathogens, and hormones may be central to understanding amphibian declines [17]. For example, whether the prevalence of disease associated with amphibian declines is the result of increased pathogen prevalence and/or virulence, or physiological responses to deteriorating environmental conditions (i.e., exposure to man-made chemicals) that decrease host resistance and/or behaviors that affect parasitism is still unclear and presents a major challenge in amphibian decline biology [8, 17, 21, 97]. In the present study, we assessed the potential links among environmental contaminants, endocrine physiology, and nematode abundances in *S*. *sordida* along an elevational gradient in Costa Rica–a region with well-documented cases of amphibian population declines in montane regions [8] that are at high risk of exposure to environmental contaminants [51, 52]. We detected at least one of the 31 measured environmental contaminants in approximately half of the male *S*. *sordida* sampled. Our analyses focused on organochlorines, a carbamate (carbaryl), two organophosphates (malathion and chlorpyriphos), the benzidine herbicide atrazine, and the chlorophenoxy herbicide 2,4 D (see S1 Appendix). These contaminants have potentially spread to every region of the globe via precipitation [25], have been used either historically or currently in Costa Rica [83, 98], and have been linked to amphibian declines [11, 17, 99]. Nonetheless, we found no evidence that montane populations were more likely to possess any of the measured contaminants than low elevation populations. However, we cannot eliminate the possibility that other unmeasured contaminants may be more prevalent in montane regions or act synergistically to affect populations [13, 99].

We also found no evidence that high elevation populations possessed higher circulating glucocorticoid levels than low elevation populations, suggesting that montane *S*. *sordida* populations were not likely to be immunocompromised as a result of chronic stress. Surprisingly, however, montane populations had lower androgen levels, more species of nematodes, and higher nematode abundances than low elevation populations. Although these results suggested that nematodes negatively affected androgen levels in high elevation populations, further analysis revealed no evidence of an inverse relationship between nematode abundances and circulating androgen levels. The lack of a negative association between these two variables is perplexing because nematodes appeared to contribute significantly to the body mass of *S*. *sordida* (e.g., nematode abundance was positively related to estimates of body condition) and often take a toll on the general health of their host (see [19] for review in amphibians). However, macroparasites can also alter the endocrine physiology of their host, which can include increased production of sex steroids [100]. Alternatively, hormones can alter behaviors that influence susceptibility to parasites [67, 101].

Androgen and baseline glucocorticoid concentrations often vary geographically in vertebrates [102]. In birds, for example, androgen level is often positively correlated with latitude and/or elevation [103]. In amphibians, testosterone level is generally not related to elevation, while corticosterone level is often negatively related to elevation [102]. However, both testosterone level and corticosterone level are often negatively correlated with the length of the breeding season in amphibians, and breeding season length is often shorter at high elevations and latitudes [102]. Populations at higher altitudes and latitudes often have a narrower window of opportunity for mating because environmental conditions potentially constrain the length of the breeding period. Presumably, shorter breeding periods increase interactions among males that drive elevations in circulating androgen and corticosterone levels [102]. We found little evidence to support these hormonal patterns or predictions in *S*. *sordida*. For example, high elevation populations actually had lower levels of androgens than low elevation populations and corticosterone level was not significantly correlated with elevation.

Circulating glucocorticoids and androgens are often highly labile and subject to modulation via environmental and/or social conditions. For example, androgen levels can change dramatically with weather conditions [104], social interactions [105], and seasonally [106]. These factors may have contributed to lower androgen levels in high elevation populations of *S*. *sordida* if high and low elevation populations were sampled at different stages of the reproductive period. For example, high elevation populations may have been nearer to the beginning or end of their reproductive period when androgen levels are expected to be lower than during peaks in reproductive activity [107]. Temporal patterns of androgen production can vary considerably among anuran species [81, 82,108–110] with peaks in androgen level often corresponding to periods of rainfall [81, 111]. In the present study, breeding activity of *S*. *sordida* and rainfall patterns were very similar among high and low elevation sites as were developmental stages of tadpoles and basin building associated with egg deposition [see 77, 79]. We also attempted to select study sites with similar chorus densities because of its potential effect on circulating hormone levels in anurans [112]. Importantly, if males in low elevation sites with higher androgen levels were at a peak in their reproductive period and high elevation populations were not, then we would also expect to see higher glucocorticoid levels in low elevation populations because the two steroids are often positively correlated in calling males [81, 107, 110]. However, we found weak evidence for differences in CORT levels for high versus low elevation populations despite higher androgen levels in low elevation populations. Nonetheless, we cannot eliminate the possibility that elevational differences in androgen levels in *S*. *sordida* were attributable to differences in the stage of breeding or other environmental conditions that may have influenced circulating androgen levels. Further work is needed to determine if androgens are consistently lower in montane regions and whether such differences pose threats to high elevation populations.

## Conclusions

Because there is increasing evidence linking environmental contaminants, endocrine disruption, and pathogens to amphibian declines, there is an urgency to understand the interrelationships among these factors, particularly in the context of montane populations that are especially prone to decline. While the current study found differences in the endocrinology (e.g., androgen levels) and nematode parasite abundances in high and low elevation populations of *S*. *sordida*, there was no evidence to suggest that these differences were related to contaminants, nor was there evidence linking the prevalence of parasites at high elevations to endocrine factors that potentially compromise immunity. Nonetheless, lower androgen levels and higher parasite abundances in montane populations of *S*. *sordida* could pose threats to long-term health and warrants further study.

## Supporting information

S1 Appendix Contaminants examined from liver samples of *S*. *sordida*. Analysis consisted mostly of organochlorines unless noted otherwise.
(DOCX)

S1 Fig Circulating hormone levels in *S*. *sordida* from northern and southern latitude populations and high and low elevation sites. A) Progesterone, B) dihydrotestosterone, C) testosterone, D) estradiol and E) corticosterone. Numbers on abscissa represent sites depicted in Fig 1: 1) Bijagual Field Station (n = 10), 2) Bijagual Creek (n = 10), 3) Bijagualito Creek (n = 10), 4) Quebrada Bonita (n = 7), 5) Quebrada Pita (n = 10), 6) Rio Tarcolitos (n = 11), 7) Quebrada Cantarrana (n = 7), 8) Rio Jaba (n = 11), 9) Rio Sabalito (n = 5), 10) Rio Sabalito-Juntas (n = 9), 11) Quebrada Pavo (n = 1), 12) Rio Coto Colorado (n = 13), 13) Quebrada Minas (n = 5), 14) Rio Oro (n = 10).
(PPTX)

S1 Table Top 5 megaBLAST matches for each nematode parasite 18S sequence from *S*. *sordida*. Column headers are defined as follows: SMSO Field No. = Collection number; Morphospecies = Type identified in the field; Locality = Collection site; Site Type = High or Low elevation site; Region = North or South latitude; Position Found in *Smilisca* = Location in body where nematode was found; Bit-score = megaBLAST bit score; % Identical Sites = Percent of identical sites between query sequence and BLAST hit; % Pairwise Identity = Percent of identical sites between pairwise alignment between query and subject sequence; E-value = Expectation value of the probability to obtain score after correction for multiple testing; Grade = Percentage calculated by combining weighted query, e-value, and identity value hits; Accession = Genbank accession number of subject sequence; Organism = Organism of subject sequence; Description = Full description of hit sequence.
(CSV)
