# Supplementary material for: Assessing the links among environmental contaminants, endocrinology, and parasites to understand amphibian declines in montane regions of Costa Rica
Source: PLoS One. 2018 Jan 11;13(1):e0191183. doi: 10.1371/journal.pone.0191183 (PMC5764372; doi:10.1371/journal.pone.0191183)
Supplement: S1 Appendix — Analysis consisted mostly of organochlorines unless noted otherwise. (DOCX) [file pone.0191183.s001.docx]

1. Acetochlor
2. 1,2,4,5-tetrachlorobenzene
3. 1,2,3,4-tetrachlorobenzene
4. pentachlorobenzene
5. alpha-HCH
6. hexachlorobenzene
7. beta-HCH
8. gamma-HCH
9. heptachlor
10. aldrin
11. heptachlor epoxide
12. oxychlordane
13. trans(gamma)-chlordane
14. cis(alpha)-chlordane
15. alpha-endosulfan
16. trans-nonachlor
17. 4,4-DDE
18. dieldrin
19. endrin
20. beta-endosulfan
21. cis-nonachlor
22. 4,4-DDD
23. 4,4-DDT
24. photomirex
25. methoxychlor
26. mirex
27. carbaryl (carbamate pesticide)
28. malathion (organophosphate pesticide)
29. chlorpyriphos (organophosphate pesticide)
30. atrazine (benzidine herbicide)
31. 2,4 D (chlorophenoxy herbicide)
