# Supplementary material for: Assessing the links among environmental contaminants, endocrinology, and parasites to understand amphibian declines in montane regions of Costa Rica
Source: PLoS One. 2018 Jan 11;13(1):e0191183. doi: 10.1371/journal.pone.0191183 (PMC5764372; doi:10.1371/journal.pone.0191183)
Supplement: S1 Fig — A) Progesterone, B) dihydrotestosterone, C) testosterone, D) estradiol and E) corticosterone. Numbers on abscissa represent sites depicted in Fig 1: 1) Bijagual Field Station (n = 10), 2) Bijagual Creek (n = 10), 3) Bijagualito Creek (n = 10), 4) Quebrada Bonita (n = 7), 5) Quebrada Pita (n = 10), 6) Rio Tarcolitos (n = 11), 7) Quebrada Cantarrana (n = 7), 8) Rio Jaba (n = 11), 9) Rio Sabalito (n = 5), 10) Rio Sabalito-Juntas (n = 9), 11) Quebrada Pavo (n = 1), 12) Rio Coto Colorado (n = 13), 13) Quebrada Minas (n = 5), 14) Rio Oro (n = 10). (PPTX) [file pone.0191183.s002.pptx]

## Slide 1
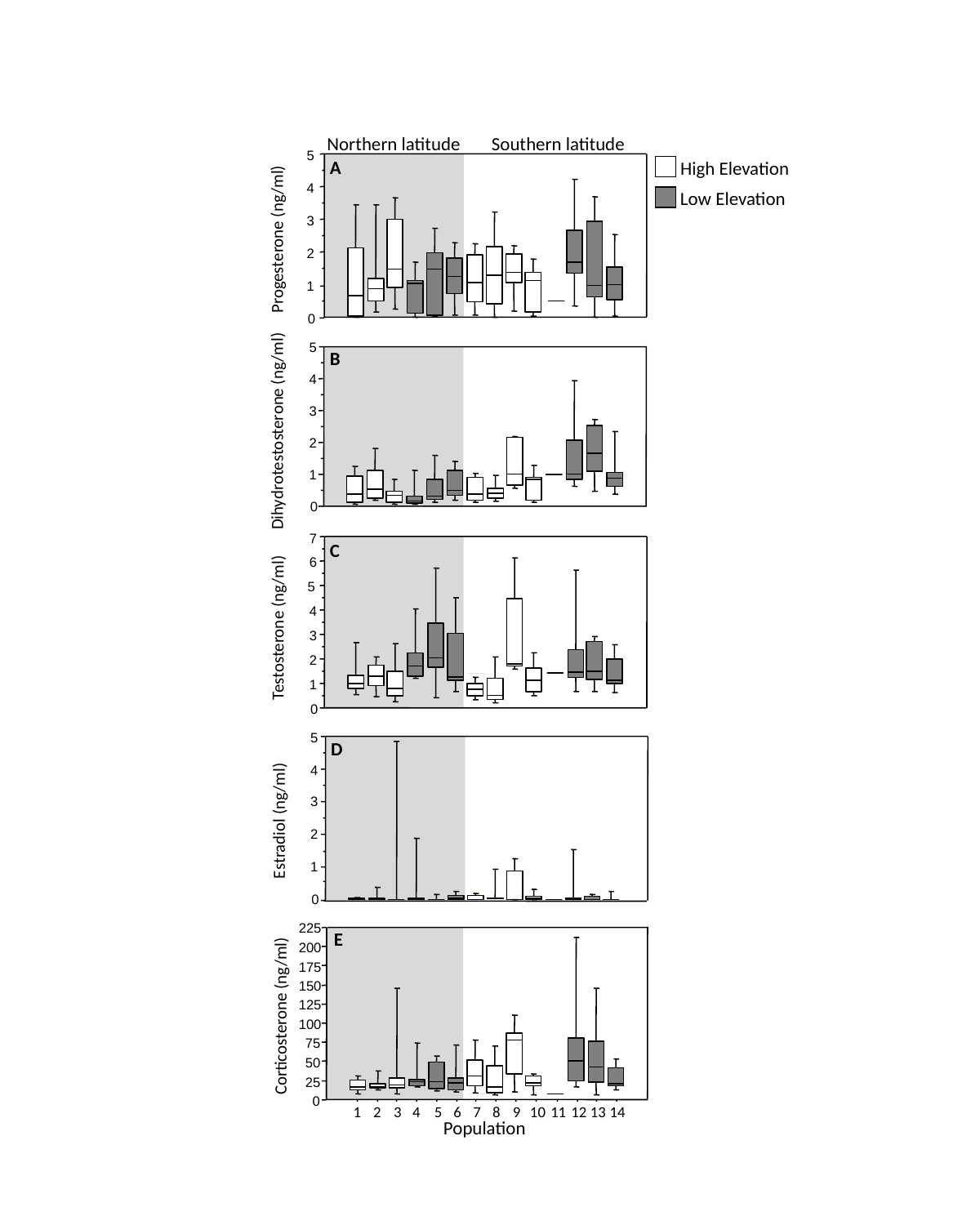

Northern latitude
Southern latitude
5
A
4
3
Progesterone (ng/ml)
2
1
0
High Elevation
Low Elevation
5
B
4
3
2
1
0
Dihydrotestosterone (ng/ml)
7
C
6
5
4
Testosterone (ng/ml)
3
2
1
0
5
4
3
2
1
0
D
Estradiol (ng/ml)
225
E
200
175
150
Corticosterone (ng/ml)
125
100
75
50
25
0
1
2
3
4
5
6
7
8
9
10
11
12
13
14
Population
